# Regulation of podosome formation, microglial migration and invasion by Ca^2+^-signaling molecules expressed in podosomes

**DOI:** 10.1186/1742-2094-9-250

**Published:** 2012-11-17

**Authors:** Tamjeed A Siddiqui, Starlee Lively, Catherine Vincent, Lyanne C Schlichter

**Affiliations:** 1 Toronto Western Research Institute, 399 Bathurst Street, Toronto, Ontario, M5T 2S8, Canada; 2 Department of Physiology, University of Toronto, 1 King's College Circle, Toronto, Ontario, M5S 1A8, Canada

**Keywords:** Small-conductance Ca^2+^-activated K^+^ channel, Ca^2+^-binding molecules, Calmodulin, Cell adhesion, Cell migration and invasion, Extracellular matrix degradation, lonized Ca^2+^ binding adapter protein 1, Orai1/CRAC, SK3, STIM1

## Abstract

**Background:**

Microglia migrate during brain development and after CNS injury, but it is not known how they degrade the extracellular matrix (ECM) to accomplish this. Podosomes are tiny structures with the unique ability to adhere to and dissolve ECM. Podosomes have a two-part architecture: a core that is rich in F-actin and actin-regulatory molecules (for example, Arp2/3), surrounded by a ring with adhesion and structural proteins (for example, talin, vinculin). We recently discovered that the lamellum at the leading edge of migrating microglia contains a large F-actin-rich superstructure (‘podonut’) composed of many podosomes. Microglia that expressed podosomes could degrade ECM molecules. Finely tuned Ca^2+^ signaling is important for cell migration, cell-substrate adhesion and contraction of the actomyosin network. Here, we hypothesized that podosomes contain Ca^2+^-signaling machinery, and that podosome expression and function depend on Ca^2+^ influx and specific ion channels.

**Methods:**

High-resolution immunocytochemistry was used on rat microglia to identify podosomes and novel molecular components. A pharmacological toolbox was applied to functional assays. We analyzed roles of Ca^2+^-entry pathways and ion channels in podosome expression, microglial migration into a scratch-wound, transmigration through pores in a filter, and invasion through Matrigel™-coated filters.

**Results:**

Microglial podosomes were identified using well-known components of the core (F-actin, Arp2) and ring (talin, vinculin). We discovered four novel podosome components related to Ca^2+^ signaling. The core contained calcium release activated calcium (CRAC; Orai1) channels, calmodulin, small-conductance Ca^2+^-activated SK3 channels, and ionized Ca^2+^ binding adapter molecule 1 (Iba1), which is used to identify microglia in the CNS. The Orai1 accessory molecule, STIM1, was also present in and around podosomes. Podosome formation was inhibited by removing external Ca^2+^ or blocking CRAC channels. Blockers of CRAC channels inhibited migration and invasion, and SK3 inhibition reduced invasion.

**Conclusions:**

Microglia podosome formation, migration and/or invasion require Ca^2+^ influx, CRAC, and SK3 channels. Both channels were present in microglial podosomes along with the Ca^2+^-regulated molecules, calmodulin, Iba1 and STIM1. These results suggest that the podosome is a hub for sub-cellular Ca^2+^-signaling to regulate ECM degradation and cell migration. The findings have broad implications for understanding migration mechanisms of cells that adhere to, and dissolve ECM.

## Introduction

In the healthy adult CNS, microglia constantly survey the environment, extending and retracting their ramified processes, but without overall cell displacement [1,2]. Microglia migrate during the perinatal period of development, and after acute injury in the adult, they activate and move to the damage site (reviewed in [3-5]). In general, cell migration requires cleavage of cell-cell or cell-matrix interactions [6]. Little is known about how microglia navigate through the densely packed brain tissue with its unique extracellular matrix (ECM). Outside the CNS, two related structures identified in several cell types have the unique ability to both adhere to and degrade ECM molecules. Podosomes (non-cancer cells) and invadopodia (cancer cells) differ from other cell adhesion structures in this dual functionality, and in their composition, architecture and dynamics (recently reviewed in [7-9]).

We recently found that in microglia, the lamellum at the leading edge contains many podosomes that spontaneously organize into super-structures we call ‘podonuts’ [10]. Microglial podosomes express several hallmark features of podosomes in other cells. They are tiny (<1 μm diameter) punctate structures at the ventral cell surface, with F-actin in the core surrounded by a ring of the plaque protein, talin. Microglial podosomes are also enriched in phosphotyrosine residues, and in three tyrosine-kinase regulated molecules that have only been reported in a few cell types. These are Tks5 (tyrosine kinase substrate with five Src homology 3 domains), tyrosine phosphorylated caveolin-1, and nicotinamide adenine dinucleotide phosphate oxidase 1 (Nox1) [10]. Microglia that expressed podonuts were able to degrade the ECM components, fibronectin and Matrigel™. Thus, these structures can potentially aid in migration through brain tissue. Despite their molecular complexity, podosomes are highly dynamic with lifetimes of 2 to 20 minutes [8,11]. However, mechanisms that regulate their rapid turnover are not well understood.

Ca^2+^ entry regulates several processes involved in cell migration, including cell-substrate adhesion and contraction of the actomyosin network [12,13]. In immune cells, store-operated Ca^2+^ entry is predominantly mediated by Orai1, which is the pore-forming subunit of the Ca^2+^-release activated Ca^2+^ (CRAC) channel [14,15]. We previously characterized the CRAC current in rat microglia. It is activated by store depletion, highly selective for Ca^2+^, and is strongly inward-rectifying, which results in much greater Ca^2+^ influx at hyperpolarized membrane potentials [16,17]. In non-excitable cells, including microglia, small-conductance Ca^2+^-activated K^+^ (SK) channels are well designed to respond to a slight elevation in intracellular Ca^2+^ and maintain a hyperpolarized membrane potential. SK channel opening does not require depolarization. Instead, they open when Ca^2+^ (K_d_ 200 to 800 nM) [18,19] interacts with calmodulin that is bound to the channel’s proximal C-terminus [20-22]. We previously showed that rat microglia express SK3 (KCa2.3/*KCNN3*) and SK4 (IK1/KCa3.1/*KCNN4*) channels. Both channels contribute to lipopolysaccharide-induced microglial activation (through p38 MAPK) and their consequent ability to kill neurons [23,24]. Of note, we observed that SK3 expression increased in activated microglia that had migrated into stroke lesions *in vivo *[24]. Therefore, we examined whether microglial podosomes contain SK3 and its obligatory subunit, calmodulin. Then, we conducted experiments to test the hypothesis that podosome formation and microglial migration are regulated by Ca^2+^ entry through CRAC channels. Based on the results, we further hypothesized that podosomes contain Orai1 and its accessory molecule, STIM1 (stromal interaction molecule 1).

## Methods

### Cells

All procedures on animals were approved by the University Health Network Animal Care Committee, in accordance with guidelines from the Canadian Council on Animal Care. Microglia cultures were prepared from 1- to 2 day-old Sprague–Dawley rat pups (Charles River, St.-Constant, PQ, Canada) using our standard protocols, which yield ≥99% purity [23,24]. In brief, after removal of the meninges, the brain was dissected, minced in cold Minimal Essential Medium (MEM; Invitrogen, Carlsbad, CA, USA), centrifuged (300 × g, 10 min) and re-suspended in MEM supplemented with 10% fetal bovine serum (FBS; Wisent, St-Bruno, PQ, Canada), and 0.05 mg/ml gentamycin (Invitrogen). The dissociated cells were then seeded in 75 cm^2^ flasks and incubated at 37°C and 5% CO_2_. After 48 hr, the medium was replaced with fresh medium to remove cellular debris and non-adherent cells, and after a further 4 to 5 days in mixed culture, microglia were harvested by shaking the flasks for 2 to 4 hr on an orbital shaker at 65 rpm (37°C, 5% CO_2_). After centrifuging the microglia-rich supernatant (300 × g, 10 min), the cell pellet was re-suspended in fresh MEM (with 2% FBS), and seeded onto UV-irradiated 15 mm glass coverslips (Fisher Scientific, Ottawa, ON, Canada) at 50,000 or 60,000 cells per coverslip in 12-well plates, and cultured for 1 to 2 days in MEM (2% FBS). Importantly, we find that under these growth conditions, their starting state is relatively resting [25].

### Roles of Ca^2+^ entry in podosome formation

Isolated microglia were cultured for 24 to 48 hr and then the tissue culture medium was replaced with standard bath solution containing (in mM): 125 NaCl, 5 KCl, 1 MgCl_2_, 1 CaCl_2_, 5 D-glucose and 10 HEPES, and adjusted to pH 7.4 with NaOH. The osmolarity was measured with an Advanced Micro Osmometer (Model 3300; Advanced Instruments Inc, Norwood, MA, USA), and adjusted to 285 to 300 mOsm with 0.5 to 2 g/l sucrose. Five channel inhibitors were tested. Stock solutions in DMSO were made for 2-APB (2-aminoethyl diphenylborinate; Sigma-Aldrich, St. Louis, MO, USA), BTP2 (N-{4-[3,5-bis(trifluoromethyl)-1H-pyrazol-1-yl]phenyl}-4-methyl-1,2,3-thiadiazole-5-carboxamide; EMD Millipore Calbiochem, San Diego, CA, USA) and NS8593 (Sigma-Aldrich). Stock solutions in ddH_2_O were made for gadolinium (III) chloride (GdCl_3_; Sigma-Aldrich) and spermine tetrahydrochloride (EMD Millipore Calbiochem). For Ca^2+^-free bath solutions (0 Ca^2+^), the tissue culture medium was replaced with standard bath solution lacking CaCl_2_ and with 1 mM ethyleneglycol-bis(β-aminoethyl)-N,N,N’,N’-tetraacetic acid (EGTA). Control cells were maintained in standard bath solution under identical experimental conditions. Bath solutions were sterilized by filtering through 0.2 μm filters, and all treatments were performed at 37°C for 30 min. Before fixing treated cells for immunocytochemistry, cells were washed once with sterile phosphate-buffered saline (PBS). Large rings of podosomes (podonuts) were counted from three random fields of cells on each cover slip, and averaged over several microglia cultures prepared from different animals.

### Immunocytochemistry and cell labeling

The methods were similar to our recent paper [10]. Microglia were seeded at 50,000 to 60,000/glass cover slip and cultured for 2 to 3 days in MEM with 2% FBS. They were then fixed in 4% paraformaldehyde (Electron Microscopy Sciences, Hatfield, PA, USA) at room temperature for 15 min. The cells were permeabilized with 0.2% Triton X-100 for 5 min and washed in PBS (3×, 5 min each). Non-specific antigens were blocked with 4% donkey serum for 1 hr. All antibodies (Table 1) were diluted in 2.5% donkey serum and centrifuged before use (8200 × g, 10 min) to precipitate any aggregated antibody. Microglia were incubated with one or two primary antibodies overnight at 4°C, washed (3×, 5 min each) and blocked with 4% donkey serum for 1 hr. They were incubated with an anti-rabbit, anti-mouse or anti-goat secondary antibody (all raised in donkey) for at least 1 hr, and then washed (3×, 10 min each). Negative controls were prepared using the same protocol, but omitting each primary antibody. We previously validated the SK3 antibody by comparing staining in SK3-free CHO cells versus those transfected with SK3 [24]. The other primary antibodies have all been used in the literature and assumed to be specific. F-actin was visualized by incubating the cells (15 min, room temperature) with Alexa Fluor™ 488-conjugated phalloidin (1:50 in blocking solution, Invitrogen). Occasionally, microglia were co-labeled with FITC-conjugated tomato lectin (Sigma-Aldrich), which binds to N-acetyl-lactosamine residues on the microglia surface [26]. In this case, after applying secondary antibody and washing, microglia were incubated with tomato lectin (1:500 in PBS with 2.5% donkey serum; 15 min, room temperature) and then processed as described above. Cell nuclei were labeled with 4’,6-diamidino-2-phenylindole (DAPI; 1:3000 in PBS, 5 min; Invitrogen). After washing (3×, 5 min each), cells on coverslips were mounted on glass slides with 50% glycerol in PBS (which produced the lowest background), VectaShield™ (Vector Labs, Burlington, CA, USA) or Dako mounting medium (Dako, Glostrup, Denmark). Dako mounting medium yielded more stable signals for longer imaging (Z-stacks). For Orai1 and CaM staining, we used an antigen retrieval step after fixation. Cover slips were microwaved on medium power for 3 min in citrate buffer, cooled in buffer and washed with PBS. Antigen retrieval is used to unmask epitopes that are obscured by crosslinking [27].

**Table 1:** Antibodies

**Primary antibodies (species and type)**	**Dilution**	**Supplier**
α-Tubulin (mouse monoclonal)	1:1000	Abcam (Cambridge, MA, USA)
Arp2 (rabbit polyclonal)	1:100	Santa Cruz Biotechnology (Santa Cruz, CA, USA)
CaM (rabbit monoclonal or mouse monoclonal)	1:200	Abcam
Iba-1 (rabbit polyclonal)	1:1000	Wako Chemicals (Richmond, VA, USA)
Orai-1 (goat polyclonal)	1:100	Santa Cruz Biotechnology
SK3 (rabbit polyclonal)	1:200	Alomone (Jerusalem, Israel)
STIM1 (rabbit polyclonal)	1:100	Sigma-Aldrich (St. Louis, MO, USA)
Talin1/2 (mouse monoclonal)	1:100	Abcam
TRPM7 (goat polyclonal)	1:200	Santa Cruz Biotechnology
Vimentin (mouse monoclonal)	1:1000	Millipore (Billerica, MA, USA)
Vinculin (mouse monoclonal)	1:200	Sigma-Aldrich
**Secondary antibodies**		
DyLight 488 or 594 (donkey anti-rabbit)	1:500	Jackson Immunoresearch (West Grove, PA, USA)
DyLight 488 or 594 (donkey anti-mouse)	1:500	Jackson Immunoresearch
DyLight 488 (donkey anti-goat)	1:500	Jackson Immunoresearch

Images were acquired with an Axioplan 2 wide field epifluorescence microscope equipped with an Axiocam HRm digital camera, and were analyzed with Axiovision 4.6 software (all from Zeiss, Toronto, ON, Canada) or ImageJ [28]. For many images, we acquired Z-stacks of high-magnification epifluorescence images through the entire cell in 200 nm increments. These images were deconvolved using a theoretical point spread function and the constrained iterative maximum likelihood algorithm in Axiovision 4.7 software (Zeiss). Deconvolution reduces noise and distortions introduced during image acquisition. It uses information about the optical system (for example, type of objective lens, refractory index of the immersion medium) to calculate a point spread function of light above and below the plane of focus (reviewed in [29]). Cell auto-fluorescence and non-specific staining were subtracted by using the same imaging and acquisition settings on cells exposed to a secondary antibody alone. When constructing Z-stacks, the automated correction algorithm was used to compensate for fluorescence decay during repeated exposures.

### Migration, substrate degradation and invasion assays

#### Live imaging

Microglia were plated at 60,000 cells/dish in 35 mm glass bottom culture dishes (MatTek Corporation, Ashland, MA, USA), and cultured for 2 days in MEM with 2% FBS. Cells were imaged for up to 1 hr using a Zeiss Axiovert 200M microscope, ORCA-ER camera (Hamamatsu Corporation, Bridgewater, NJ, USA), Axiovision software (Zeiss) and Neue LiveCell™ stage top incubator (Pathology Devices, Westminster, MD, USA) to maintain a temperature of 37°C and 5% CO_2_.

### *Scratch wound*

Microglia were seeded at 50,000 to 60,000 cells per 15 mm glass cover slip, and cultured in MEM with 2% FBS until approximately 80% confluent (1 to 2 days). The resulting monolayer of microglia was scratched with a sterile 200 μl pipette tip, with or without addition of a channel blocker (50 μM 2-APB, 5 μM GdCl_3_, 10 μM BTP2). The cells were incubated for 24 hr (37°C, 5% CO_2_), fixed and permeabilized as described above (Immunochemical analysis), and stained with FITC-conjugated tomato lectin (1:500, 15 min; Sigma) and DAPI (1:3000 in PBS, 5 min). Five random fields along the border of the scratch were imaged at 10× magnification using a confocal microscope (LSM 510 META; Carl Zeiss, Jena, Germany) and saved. The number of microglia infiltrating into the wound was quantified by counting all the tomato lectin-positive cells within the scratch region. At least five individual cultures were used to calculate the mean migration, and results were normalized to control (untreated) microglia.

#### Transmigration

Microglia were suspended in MEM containing 2% FBS, and 150 μl of cell suspension (2×10^4^ cells) was added to the upper well of each Transwell™ insert (VWR, Mississauga, ON, Canada), which bore an uncoated filter with 8 μm-diameter holes. The lower well contained only the medium (MEM with 2% FBS). After 30 min, a channel inhibitor (50 μM 2-APB, 5 μM GdCl_3_, 10 μM BTP2, 7 μM NS8593) was added to the upper well, and then incubated for 24 hr (37°C, 5% CO_2_). The remaining microglia on the upper side were removed with a Q-tip^TM^. To quantify the number of microglia that had transmigrated to the underside, the filter was fixed in 4% paraformaldehyde, rinsed with PBS, stained with 0.3% crystal violet for 1 min, and again rinsed with PBS. Microglia on the underside of each filter were counted in five fields of view per filter at 40× magnification using an Olympus CK2 inverted microscope (Olympus, Tokyo, Japan). At least three individual cultures were used, and results were normalized to control (untreated) microglia.

#### Substrate degradation

The standard assay for studying ECM degradation by podosomes (and invadopodia) employs fluorescent-labeled substrate (usually fibronectin or gelatin), either coated directly on glass coverslips or on a layer of gelatin. ECM degradation is then monitored as loss of substrate fluorescence. We labeled bovine fibronectin (Sigma-Aldrich, Oakville, ON, Canada) using the Alexa Fluor™ 488 Protein Labeling Kit (Invitrogen), after which the conjugated protein was separated from unconjugated dye on a column. Purified, labeled fibronectin was diluted in PBS (approximately 2 μg/ml), added to glass cover slips (150 μl/cover slip) and incubated overnight at 37°C. After the solution was aspirated off, microglia were seeded onto the fibronectin-coated cover slips (50,000 cells/cover slip) and incubated for 24 hr. Fixation and staining then proceeded as described above (Immunocytochemistry and cell labeling).

#### Invasion

Microglial invasion through a basement membrane type of ECM (Matrigel™, which is secreted by mouse sarcoma cells) was quantified using 24-well BioCoat Matrigel™ Invasion Chambers (BD Biosciences, Mississauga, ON, Canada). The filters, which had 8 μm-diameter holes coated with Matrigel™, were rehydrated for 1 hr at 37°C with 500 μl of medium (MEM, 2% FBS), which was then replaced with 500 μl of fresh MEM (with 2% FBS) containing 2×10^4^ microglia. The lower well of each chamber contained 500 μl of medium only (MEM, 2% FBS). After 30 min, microglia were treated with a channel inhibitor (50 μM 2-APB, 5 μM GdCl_3_, 10 μM BTP2, 7 μM NS8593) and then incubated (24 hr, 37°C, 5% CO_2_), and prepared and counted as above (Transmigration). At least three individual cultures were used to calculate the mean invasion, and results were normalized to control (untreated) microglia.

### Statistical analysis

Quantitative data are presented as mean ± standard error of the mean (S.E.M). One-way analysis of variance was followed by post-hoc Tukey’s test, and results are considered significant if *P* < 0.05.

## Results

### Microglial podosomes contain three Ca^2+^-regulated molecules not previously reported

**Figure 1:**
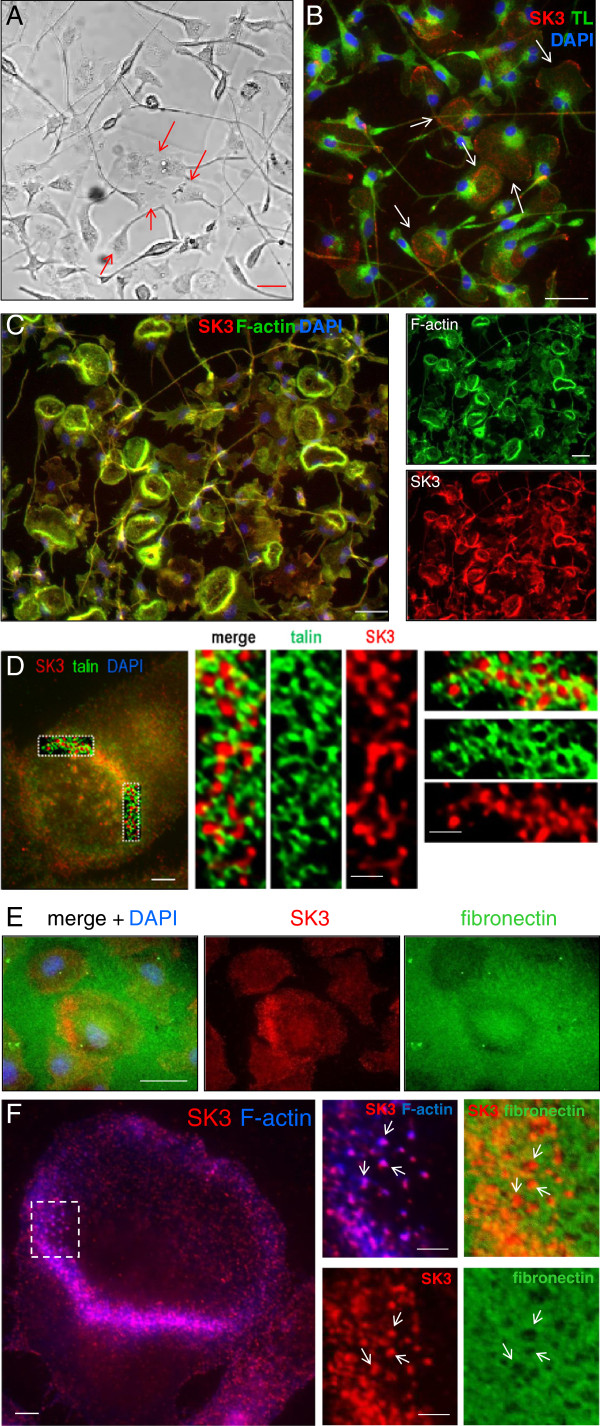
**The small-conductance Ca^2+^-activated K^+^ channel, SK3, is expressed in podosomes of rat microglia. (A)** Live imaging: Migrating microglia have a lamellum at the leading edge (see arrows). Scale bars: 40 μm in A, B, C, E. **(B)** SK3 is prevalent in a large ring within many lamellae (arrows). Microglia were labeled with tomato lectin (green), anti-SK3 antibody (red), nuclear stain, DAPI (blue throughout). **(C)** SK3-labeled rings (red) co-label for F-actin (green; Alexa Fluor™ 488-conjugated phalloidin). Color-separated images are at right. **(D)** The large SK3 ring comprises many small punctae containing the podosome ring component, talin (green). High-magnification, deconvolved images (right; boxed areas) show tiny SK3 punctae (<1 μm diameter) surrounded by talin. Scale bars, 5 μm (left), 1 μm (middle, right). **(E)** A cell-sized area of fibronectin degradation, seen as loss of Alexa Fluor™ 488 staining (green), co-localized with enriched SK3 staining (red). **(F)** Co-localization (magenta) of SK3 (red) and F-actin (Alexa Fluor™ 488-conjugated phalloidin, false-colored blue) in a ‘podonut’. Scale bar, 5 μm. Right: higher-magnification, merged and color-separated images of the boxed region showing SK3 + F-actin (merged; magenta), SK3 (red) and Alexa Fluor™ 488-conjugated fibronectin (green). Note the many tiny punctae of F-actin and SK3 staining (podosomes) and similar-sized punctae of fibronectin degradation (some examples shown by arrows). Scale bars, 1 μm.

Live-cell imaging confirmed that under the conditions in the present study, migrating microglia have a lamellum at the leading edge and a uropod at the rear (Figure 1A). We recently showed that each lamellum usually contains a large, donut-shaped, F-actin rich structure (‘podonut’) comprised of many individual podosomes [10]. Podosomes are typically recognized as a talin-rich ring surrounding a core of F-actin [7,8]. Here, we made the surprising discovery that the small-conductance Ca^2+^-activated K^+^ channel, SK3, is enriched at the leading edge of migrating microglia. SK3 was often in a single large ring in the lamellum (Figure 1B), and coincident with F-actin (Figure 1C). High-resolution deconvolution imaging of the podonut ring shows SK3 in individual podosomes (punctae <1 μm diameter), surrounded by talin (Figure 1D).

The standard test of podosome functionality is degradation of a fluorescent-labeled substrate (usually fibronectin or gelatin), which is seen as a loss of fluorescence [11,30]. The ECM component, fibronectin is not normally in the brain parenchyma, but can enter after injury [31,32]. We recently found that microglia podosomes degrade fibronectin and Matrigel™ [10]. Here, we show that when microglia were plated onto fluorescent-labeled fibronectin, its degradation could produce podonut-sized regions of reduced fluorescence (Figure 1E). Punctae of fibronectin degradation were similar to the tiny podosome-sized punctae of SK3 and F-actin (Figure 1F). [Note: The optical resolution of this *in situ* zymography is lower than for immunocytochemistry of cells plated on glass.]

**Figure 2:**
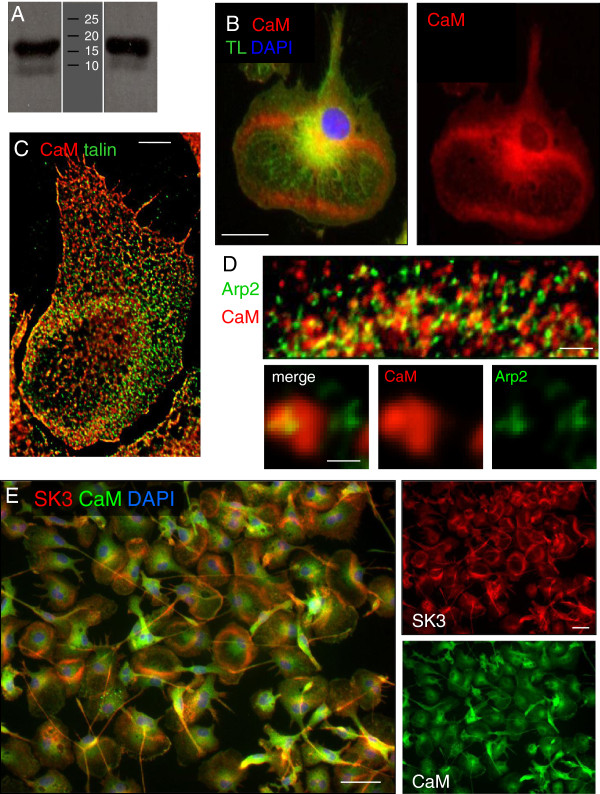
**Calmodulin (CaM) is expressed in microglia podosomes. (A)** A Western blot for CaM shows a strong band of the expected molecular weight (approximately 17 kDa) for the positive control (left lane; 50 ng recombinant human CaM protein) and in lysates from untreated cultured rat microglia (right lane). The **(B)** CaM is enriched in the podonut of lamella-bearing migrating cells. Non-deconvolved image shows a cell stained with the microglia marker, tomato lectin (TL, green), the nuclear marker, DAPI (blue), and CaM (red), which is isolated in the color-separated image at the right. Scale bar, 20 μm. **(C)** A deconvolved image of a podonut in a microglia lamellum, which shows enriched staining for CaM (red) and the podosome ring marker, talin (green). Scale bar, 5 μm. **(D)** A high-resolution, deconvolved image from a podonut ring shows CaM (red) within and surrounding the core, which is labeled with Arp2 (green). (Note: A mouse monoclonal anti-CaM antibody was used.) Scale bar, 2 μm. Lower panel: Two podosomes are shown at higher magnification; one with extensive, diffuse CaM staining surrounding Arp2. Scale bar, 1 μm. **(E)** CaM (green) and SK3 (red) co-localize in podonuts within the lamellae (nuclei are labeled with DAPI, blue). Color-separated images at the right. Scale bars, 40 μm.

CaM is essential for normal functioning of SK channels, acting as both the Ca^2+^ sensor and gate for channel opening [20,22]. Podosomes are at the ventral cell surface, and we previously discovered that CaM is required for surface membrane expression of SK4 channels [33]. This was later shown for SK2 [34] and SK3 [35]. In the healthy adult brain, CaM expression is low in microglia but is elevated in activated microglia after damage [36], when they are expected to be migratory. We hypothesized that CaM will be highly expressed in migrating microglia and present in the podosome. Western blotting showed that CaM is highly expressed in cultured, migratory rat microglia (Figure 2A). Immunostaining showed enriched CaM immunoreactivity in the podonut (Figure 2B), and co-localization with robust staining for the podosome marker, talin (Figure 2C). At high magnification, CaM was seen both adjacent to and over-lapping with the podosome core marker, Arp2 (Figure 2D). Not surprisingly, CaM co-localized with SK3 in podonuts in microglia lamellae (Figure 2E). [Note that antigen retrieval was necessary for CaM immunocytochemistry; see Methods.]

**Figure 3:**
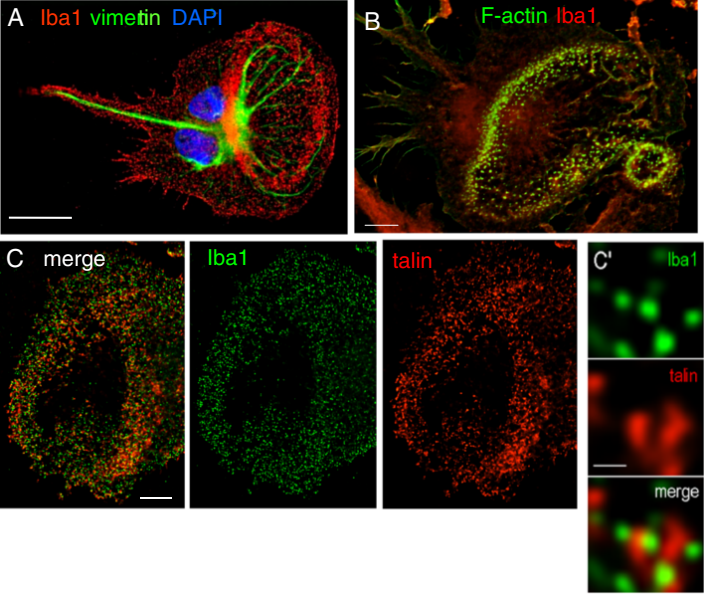
**The microglia marker Iba1 is enriched in podonuts and podosomes.** All panels show high-resolution, deconvolved images. **(A)**. Representative microglia showing strong Iba1 immunoreactivity (red) in the lamellar ring. Vimentin immunoreactivity (green) is also shown and nuclei are labeled with DAPI (blue). Scale bar, 20 μm. **(B)** A microglial cell stained for Iba1 (red) and the podosome core marker, F-actin (phalloidin; green). Scale bar, 5 μm. **(C)** Merged and color-separated images immunostained for Iba1 (green) and the podosome ring marker, talin (red). Scale bar, 5 μm. C’: High-magnification images of talin and Iba1 in individual podosomes. Scale bar, 1 μm.

In the CNS, ionized Ca^2+^ adapter molecule 1 (Iba1) is commonly used as a microglia-specific marker [37,38]. Because Iba1 is an actin-cross linking protein, we asked whether it is associated with the cytoskeleton, and specifically with podosomes. Iba1 was enriched in the podonut but did not co-localize with vimentin, which projected into the lamellum and uropod (Figure 3A). Vimentin is a major cytoskeletal component in microglia [39]. In podonuts, Iba1 was highly co-localized with F-actin (Figure 3B; yellow in the merged image). As expected, the podosome ring marker, talin was highly enriched in podonuts (Figure 3C). High-resolution, deconvolved images showed podosome-sized punctae of Iba1 (<1 μm diameter) surrounded by talin (Figure 3C’).

### Podosome formation is regulated by Ca^2+^ entry, likely through Orai1/CRAC channels

**Figure 4:**
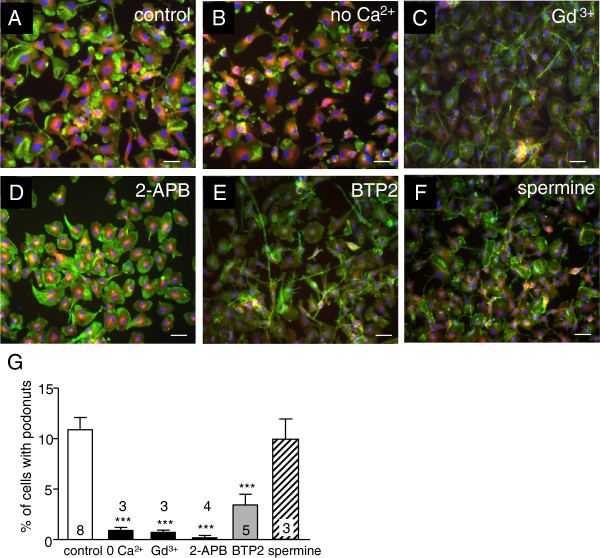
**Podonut (podosome) formation requires Ca^2+^ entry, likely through Orai1/CRAC channels. (A-F).** Microglia were fixed and stained for F-actin (phalloidin; green), talin (red), and the nuclear stain, DAPI (blue) after 30 min (37°C) incubation in: standard bath (control, **(A)**), Ca^2+^-free bath solution containing EGTA **(B)**, 5 μM gadolinium (Gd^3+^, **(C)**), 50 μM 2-APB **(D)**, 10 μM BTP2 **(E)** or 100 μM spermine **(F)**. Scale bars, 20 μm. G**.** Percentage of microglia expressing podonuts under each condition. Cells were fixed after 30 min treatment. Podonuts were counted from three random fields on each immunostained cover slip, and averaged for cultures prepared from the number of rats indicated on each bar. Results are expressed as mean ± SEM, and ***P <0.001 indicates differences from cells in standard bath (control).

Podosomes continually turn over, with a reported lifetime of 2 to 20 min [8]. We next addressed whether microglial podosomes depend on a specific route of Ca^2+^ entry. The number of cells bearing a podonut was quantified after incubation (30 min, 37°C) in bath solution with or without compounds known to affect Ca^2+^ entry in these cells. Microglia were fixed at 30 min and podonuts were visualized by staining for F-actin and talin. Representative images are shown in Figure 4A to 4F, and the proportion of cells with a podonut is summarized in Figure 4G.

Omitting external Ca^2+^ reduced podonut prevalence by more than tenfold: from 10.9 ± 1.2% of cells in control solution (Figure 4A,G) to 1.0 ± 0.2% in Ca^2+^-free solution (Figure 4B,G). The broad-spectrum Ca^2+^ channel blocker, 5 μM Gd^3+^, decreased podonut prevalence to a similar degree: to 0.7 ± 0.2% of cells (Figure 4C,G). Rat microglia express several Ca^2+^-permeable transient receptor potential (TRP) channels, including TRP melastatin 7 (TRPM7) [17,40]. TRPM7 can be blocked by 2-APB ([17]; and see Discussion), and we found that 50 μM 2-APB nearly abolished podonuts (0.2 ± 0.2% of cells; Figure 4D,G). Importantly, this concentration of 2-APB also blocks the highly Ca^2+^-selective CRAC channel produced by the pore-forming subunit, Orai1. We previously showed that CRAC is a major component of store-operated Ca^2+^ entry (SOCE) in rat microglia, and is effectively blocked by 2-APB without toxicity [17]. Therefore, we next tested BTP2, a more selective CRAC channel blocker with reported IC_50_ values ranging from 0.1 to 2.2 μM [41,42]. At 10 μM, BTP2 decreased podonut prevalence to 3.5 ± 1.0% of cells (Figure 4E,G). At 1 μM, there was no inhibition by BTP2 (data not shown). This result rules out the main potential side effect of BTP2. That is, Ca^2+^-entry through TRPM4 channels in lymphocytes was enhanced by low nanomolar concentrations of BTP2 (IC_50_ approximately 100-fold lower than for CRAC) [42]. This was a concern because TRPM4 is expressed in murine microglia [43]. Finally, to further distinguish between CRAC and TRPM7 channels, we applied spermine. We previously showed that the concentration used effectively blocks TRPM7 in rat microglia, without toxicity [40]. Podonut prevalence was not reduced by 100 μM spermine; it remained at 9.9 ± 2.0% of cells (Figure 4F,G). This provides evidence against non-specific effects or toxicity of spermine. Together, these results show that podosomes require Ca^2+^ entry, most likely through CRAC channels.

**Figure 5:**
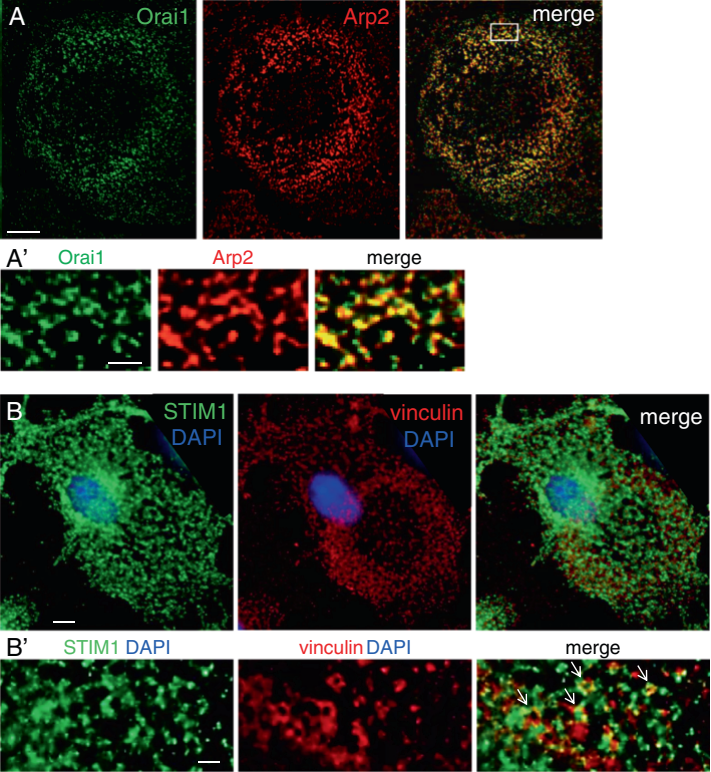
**Microglial podosomes are enriched in the CRAC/Orai1 channel and closely associated with STIM1. All panels show high-resolution, deconvolved images. (A)** The podonut region of a representative microglial cell is stained for Orai1 (green) and the podosome core marker, Arp2 (red); merged image is at the right. Scale bar, 5 μm. **(A’)** High-magnification images from the boxed region show strong co-localization of Orai1 and Arp2 in podosome cores. Scale bar, 1 μm. **(B)** Color-separated and merged images of a microglial cell stained for STIM1 (green), vinculin (red), and the nuclear stain, DAPI (blue). Note the vinculin enrichment in the podonut. Scale bar, 5 μm. **(B’)** High-magnification images from a different cell show proximity of STIM1 and vinculin, and several examples of co-localization (arrows). Scale bar, 1 μm.

CRAC current is produced by the pore-forming subunit, Orai1, and requires transient interaction with STIM1, a Ca^2+^ sensor protein that is primarily localized to the endoplasmic reticulum (ER) membrane (recently reviewed in [44]). Orai1 immunoreactivity was highly enriched in podonuts and in the core of individual podosomes, where it co-localized with Arp2 (Figure 5A). [Note that antigen retrieval was necessary for Orai1 staining; see Methods.] As expected for an ER-associated protein, STIM1 was enriched near the nucleus, and was wide-spread throughout the cell (Figure 5B). STIM1 was also prevalent in the podonut, near the podosome ring component, vinculin. High-magnification images show a close association between STIM1 and vinculin, with some co-localization in podosome rings.

**Figure 6:**
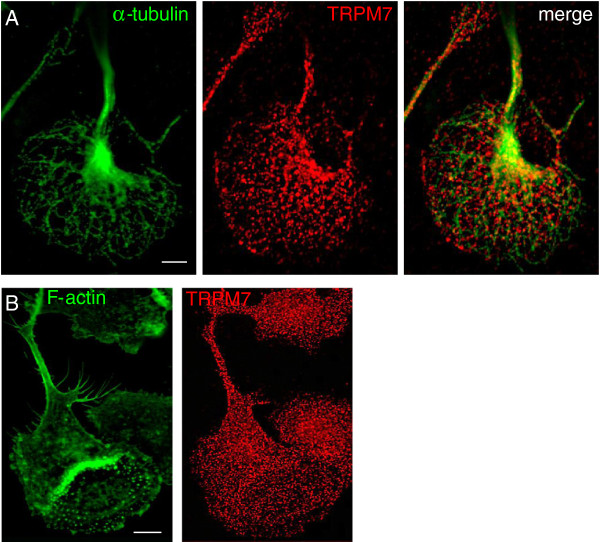
**Microglial podosomes are not enriched for the TRPM7 channel. All panels show high-resolution, deconvolved images. (A)** Color-separated and merged images of a microglial cell stained for the TRPM7 channel (red) and α-tubulin (green). **(B)** Microglia stained for TRPM7 (red) and the podosome core marker, F-actin (phalloidin, green). Scale bars, 5 μm.

An earlier study over-expressed TRPM7 in the N1E-115 neuroblastoma cell line, and showed that it induced podosomes and was present in them [45]. In contrast, in microglia, block of native TRPM7 channels with spermine had no effect (Figure 4) and the channel was not enriched in podonuts (Figure 6). Microtubules regulate podosome dynamics and localization in monocytic cells [46]. Thus, we asked whether the wide-spread, punctate TRPM7 staining was associated with α-tubulin: it was not. (Other channels that were not enriched in microglial podosomes (not shown) were TRPC3 and SK4.)

### Microglia migration and invasion involve Orai1/CRAC and SK3 channels

**Figure 7:**
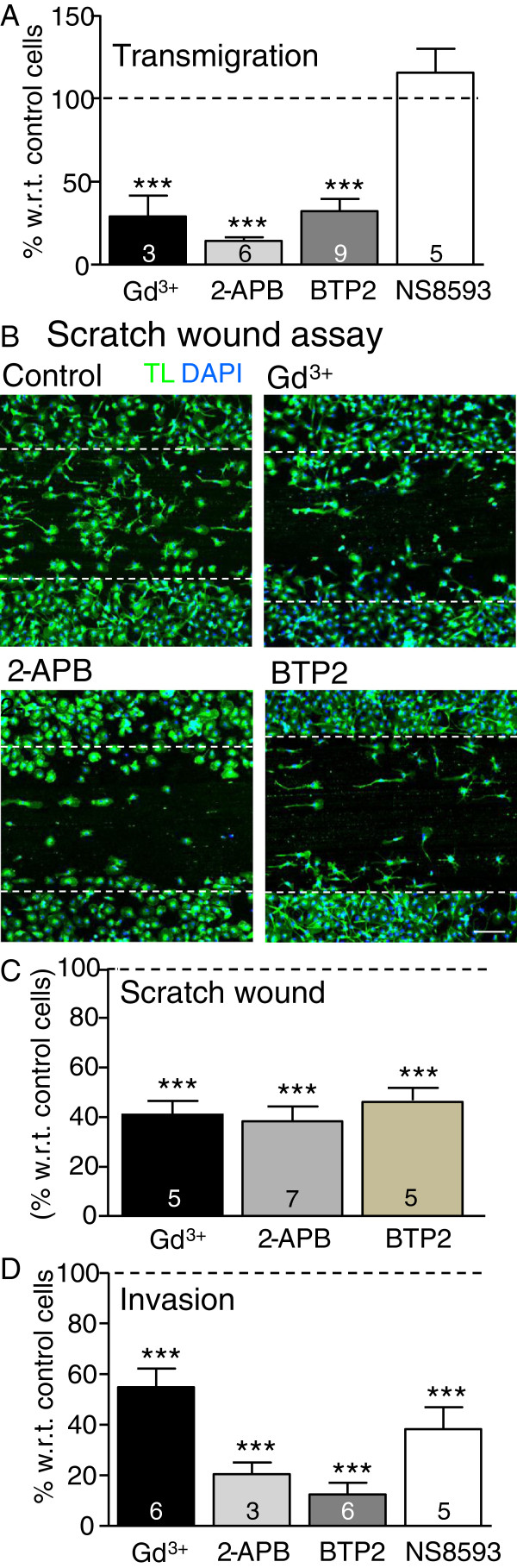
**Inhibition of microglia migration and invasion by blocking CRAC or SK3 channels. Cultured rat microglial cells were exposed for 24 hr to culture medium (MEM with 2% serum) (control), with or without a channel blocker.** CRAC blockers were 5 μM Gd^3+^, 50 μM 2-APB, 10 μM BTP2. SK3 channels were inhibited using 7 μM NS8593. **(A)** Cell transmigration across 8 μm-diameter holes in the filter of Transwell™ inserts. **(B)** Migration into a scratch wound made in an essentially confluent layer of microglia. Scale bar, 100 μm. **(C)** Invasion of microglia through filters with Matrigel™-coated 8 μm-diameter holes (BioCoat Matrigel™ Invasion chambers). For each treatment, cell counts were tallied from five random fields of view at 40× magnification (transmigration, invasion) or 10× (scratch wound), and normalized to control (untreated) microglia (100%, dashed line). The number of individual cultures used is indicated on each bar. ***P < 0.001.

Podosomes have dual roles in degrading extracellular matrix molecules and regulating cell migration (see Introduction). Therefore, we next tested whether blocking the ion channels we found in podosomes inhibits migration and substrate degradation by microglia. Three inhibitors of store-operated Ca^2+^ entry (Gd^3+^, 2-APB, BTP2) were used in 24 hr assays at the concentrations found to inhibit podosome/podonut formation (see Figure 4). Spermine, which did not inhibit podonut formation, could not be tested because it was toxic in these longer-term assays. All three Ca^2+^-channel blockers reduced three-dimensional transmigration through open 8-μm diameter holes in the filters of Transwell™ chambers. The reductions were 71% by Gd^3+^, 86% by 2-APB, 68% by BTP2 (Figure 7A) and, as discussed above, this pharmacology implicates CRAC channels.

To test the role of SK3 channels, we used the gating modulator, NS8593. This inhibitor shifts the Ca^2+^ dependence of opening of SK1, SK2 and SK3 (KCa2.1, KCa2,2 and KCa2.3) channels but does not affect the KCa3.1/SK4 channel [47]. The reported K_d_ values are < 100 nM, but because its efficacy is Ca^2+^ dependent, we used it at 7 μM to ensure that it fully inhibits SK1, SK2 and SK3. We previously reported lack of activity and contributions of SK1 and SK2 in rat microglia [24,48]; thus, we conclude that any effects of NS8593 are through SK3. NS8593 did not reduce microglia transmigration through open holes; that is, it was 115 ± 15% of the control value. This result rules out SK3 (and SK1, SK2) in this simple migration assay, and provides evidence against non-specific effects or toxicity of NS8593.

Migration in two-dimensions was examined in a scratch wound assay, in which microglia in the center of a confluent layer were removed by scratching the glass coverslip. Unlike transmigration, the scratch wound likely involves matrix molecules secreted by microglia and chemotactic factors released by the damaged cells. Migration into the scratch wound was inhibited by the CRAC channel blockers: 59% by Gd^3+^, 62% by 2-APB, and 54% by BTP2 (Figure 7B). The scratch-wound assay was also useful for examining the cell morphology. Many cells continued to express lamellae after BTP2 treatment. The SK3 blocker, NS8593, was not tested in the scratch wound because it did not affect transmigration.

Finally, microglial invasion to the underside of BioCoat™ Invasion chambers was examined. Invasion requires both migration and degradation of the basement membrane component, Matrigel™ that covers the filter holes. Given that the CRAC-channel blockers reduced migration, it is not surprising that invasion was also inhibited; that is, 45% by Gd^3+^, 80% by 2-APB, and 88% by BTP2. More notable was that invasion was reduced 62% by the SK3 inhibitor, NS8593 (Figure 7C).

Results in Figure 7 support the hypothesis that store-operated Ca^2+^ channels contribute to microglia migration and substrate degradation/invasion. The pharmacology suggests that the underlying channel is Orai1/CRAC. Further information can be gleaned by comparing the drug efficacy in transmigration (open holes) versus invasion (Matrigel™-covered holes) assays. The CRAC channel blocker, BTP2, was more effective in inhibiting invasion (by 88%) than transmigration (by 68%). The SK3 inhibitor greatly reduced invasion but did not affect microglia migration. Together, these differences suggest a crucial role for both CRAC and SK3 channels in substrate degradation.

## Discussion

Podosomes are tiny, multi-molecular structures with two key properties that can aid in cell migration through tissue. They provide anchorage and traction mediated by attachment to the ECM, and localized ECM degradation. Podosomes are distinguished by having a two-part architecture. The F-actin-rich core is surrounded by a ring containing integrins and adhesion-plaque proteins, including talin, vinculin and paxillin [49-51]. We recently discovered that the lamellae of migrating microglia contain many podosomes, often arranged into a large ring that we called a ‘podonut’ [10]. Individual podosomes were identified as tiny (< 1 μm diameter) punctae with a core with F-actin and its nucleator, Arp2/3 that is surrounded by a ring of talin. Microglia with podosomes degraded the ECM component, fibronectin. This was seen as a loss of fluorescence in cell-sized patches at low magnification, and as podosome-sized punctae (approximately 1 μm) at high magnification. The present study contributes several novel findings concerning podosome structure and regulation.

Podosomes are highly dynamic and continually assemble, mature and disassemble [7,8]. There is limited information about processes regulating their rapid turnover. Podosomes in normal cells and invadopodia in cancer cells form only after cell adhesion. A key initiating factor is thought to be cell attachment to the substrate through integrins [9,51] but this is not sufficient. Of note, myeloid-lineage cells (including our observations on microglia) are apparently unique in spontaneously forming podosomes upon cell attachment. While short-lived, podosome stability involves regulation of the actin cytoskeleton [52,53]. The podosome core contains many actin-regulating molecules. These include actin nucleators (Arp2/3 complex, formins), binding proteins (coronin, tropomyosin), filament crosslinkers (caldesmon, α-actinin) and polymerization activators (cortactin, WASp and its regulators) [8,9,50]. In addition, activation of integrins and receptor tyrosine kinases can induce intracellular cascades involving c-Src, protein kinase C and Rho- GTPases [11]. Several components of podosomes (and invadopodia) are regulated by tyrosine kinase signaling [51,54]. Thus, it is not surprising that phosphotyrosine residues are highly enriched in podosomes, including those in microglia [10].

Initially, we addressed the role of Ca^2+^ entry based on evidence that Ca^2+^ regulates cell migration and cell-substrate adhesions [12,13]. For instance, in human breast cancer cells, turnover of focal adhesions was disrupted by reducing external Ca^2+ ^[55]. That study also showed that substrate adhesion and migration were impaired by the drug, SKF96365, which blocks several Ca^2+^-permeable channels, including CRAC. Migrating cells maintain a descending intracellular Ca^2+^ gradient from the trailing uropod to the leading lamellum [13,56]. Brief, localized Ca^2+^ rises (Ca^2+^ flickers) in lamellipodia can aid cell steering [57]. For microglia, we found that Ca^2+^ influx was required for formation of podosomes (and podonuts). The pharmacological profile implicates CRAC channels in podosome formation, microglia migration into a scratch wound, transmigration through open pores, and invasion through Matrigel™. Thus, it is notable that the core of individual podosomes contained Orai1, which is the pore-forming subunit of CRAC [58]. CRAC channels open when Orai1 interacts with the ER-molecule, STIM1, which is oligomerized following depletion of intracellular Ca^2+^ stores (recently reviewed in [44]). Oligomerization is rapidly reversed (approximately 2 min) when stores are replenished, and therefore the STIM1-Orai1 interaction is transient. Podosomes are also highly dynamic, with lifetimes as short as 2 min [8,11]. Despite both processes being short-lived, we found a close association of STIM1 with podosomes, and some clear co-localization in the podosome ring. Although it would be interesting to know if podosomes transiently interact with functional CRAC channels in response to localized depletion of Ca^2+^ stores, it will be difficult to study such transient interactions.

Previous evidence linking podosomes to specific routes of Ca^2+^ entry is limited. Information derives mainly from over-expression studies and is often conflicting. Podosome formation in a neuroblastoma cell line was induced by over-expressing and activating the Ca^2+^-permeable channel, TRPM7 [45], and is consistent with a dependence on intracellular Ca^2+^. Another study addressed TRPM7 but did not examine podosomes [57]. Having found that TRPM7 produced Ca^2+^ flicker activity and was stretch-activated, the authors proposed that this channel responds to cell adhesion, traction and migration. We previously demonstrated robust expression of native TRPM7 channels in primary rat microglia [17]. In microglia, TRPM7 was not stretch-activated, and instead produced a large current under a wide range of activation conditions [40]. Here, we show that TRPM7 was not enriched in podosomes, and that blocking it did not affect podosome formation. There are also conflicting data regarding the role of Ca^2+^ in podosome formation. Two earlier studies found that elevating intracellular Ca^2+^reduced podosome numbers, but direct comparisons are difficult. One study was in a macrophage cell line transfected with the Ca^2+^-permeable channel, TRP Vanilloid 2 [59]. The second was in chicken osteoclasts under several conditions that raised intracellular Ca^2+^[60], including high extracellular K^+^ or activators of voltage-gated Ca^2+^ channels. Because microglia lack voltage-gated Ca^2+^ currents [3,61], this finding is not expected to translate.

We examined subcellular localization of the Ca^2+^-activated K^+^ channel, SK3, because of our previous findings that SK3 was increased in activated rat microglia *in vivo* and regulated their classical activation *in vitro *[24]. We surmised that SK3 likely acts by maintaining a driving force for Ca^2+^ entry through Orai1/CRAC channels. No previous reports have linked SK3 channels to podosomes. Here, we found that SK3 was highly enriched in the podosome core. Importantly, its accessory molecule, CaM, was present both within and around the podosomes. This is significant because the Ca^2+^ sensitivity of SK channel gating is conferred by CaM, which is bound to the channel’s carboxy-terminus [21,22]. SK channels open after Ca^2+^ binds to CaM [62]. Here, we found that the SK3 channel inhibitor, NS8593, did not affect migration through open holes but dramatically reduced microglia invasion through Matrigel™. Thus, we conclude that SK3 is involved in matrix degradation. SK3 has been implicated in migration of some cancer cells. In one study, an SK3-dependent membrane hyperpolarization increased the motility of melanoma cells [63]. In another, SK3 was expressed in tumor breast biopsies and a highly metastasizing mammary cancer cell line, but not in non-tumor breast tissue [12]. The latter study showed that SK3 blockers inhibited migration, depolarized the cell, and reduced intracellular Ca^2+^. We found one report on non-cancer cells. SK3 was present in lamellipodia and filopodia of neural progenitor cells [64]. Pharmacological treatments implicated the channel in formation of cellular projections, which are structures used to explore the local environment, interact with other cells and for migration.

Several other Ca^2+^-regulated molecules have been identified in podosomes but mainly in transformed cells. Caldesmon was found in Src-transformed fibroblasts [65], calponin in the A7r5 smooth-muscle cell line [66], gelsolin in Src-transformed fibroblasts and monocyte-derived cells [67], and Pyk2 in the MB1.8 osteoclast cell line [68]. We found that microglial podosomes are highly enriched in the Ca^2+^-binding molecule, Iba1, which is a marker used to identify microglia and infiltrating macrophages in the CNS [37,38]. Iba1 has not previously been reported in podosomes or invadopodia but was of interest because it cross-links actin filaments in a Ca^2+^ dependent manner [69]. Previously, Iba1 was found in membrane ruffles and phagocytic cups of MG5 cells, a microglia cell line from p53-deficient mice [70]. However, Iba1 is not characteristic of all F-actin-rich structures, and is not in filopodia or stress fibers [70]. We speculate that Iba1, which is in the core of microglial podosomes, might stabilize them by cross-linking F-actin. Other Ca^2+^-regulated actin cross-linking proteins might play a similar role. For instance, α-actinin is present in the podosome ring and core of macrophages, osteoclasts, monocytes, and Src-transformed fibroblasts [51,71-73].

Finally, we present a scheme to relate the literature on podosome formation and roles to the present study and our recent paper [10]. A key initiating factor in podosome formation is cell attachment to the substrate through integrin binding. The subsequent signaling is thought to activate Src, and promote phosphorylation and activation of substrates that include caveolin-1 [74,75] and Tks5 [76,77]. Phosphorylated Tks5 can act as an organizer, recruiting other proteins, including Nox1 [78], which is an enzyme that generates reactive oxygen species. We recently showed that Tks5 and Nox1 are constituents of microglial podosomes [10]. Src can activate PLCγ [79] and release soluble inositol-1,4,5-triphosphate (IP_3_), which evokes depletion of Ca^2+^ stores. This is followed in some cell types by interaction of STIM1 with Orai1 and activation of CRAC channels. We previously showed that store depletion activates CRAC currents in rat microglia [17]. Here, we show that Orai1 and STIM1 are both present in and around microglia podosomes. Ca^2+^ influx, most likely through CRAC channels, regulated microglia podosome formation, migration and invasion through Matrigel™. Invasion also required SK3 channels, which we discovered were present in podosomes, along with their gating molecule, CaM. We propose a working model in which localized Ca^2+^ elevation caused by CRAC channels (Orai1 + STIM1) activates Ca^2+^-dependent SK3 channels. The resulting K^+^ efflux is expected to hyperpolarize the membrane and help maintain a driving force for Ca^2+^ entry. Ca^2+^ entry is then expected to regulate multiple downstream effector molecules that contribute to cell migration.

## Conclusions

The expression of podosomes in microglia has broad implications. To carry out their functions, microglia must migrate within the brain parenchyma. This requires locally restricted ECM degradation, but without damaging brain cells. Podosomes might help microglia migrate in the developing brain, and after damage or in disease states when there is inflammation and matrix remodeling. These unique structures contain several molecules that suggest they are a hub for localized Ca^2+^ signaling to regulate both adhesion and ECM substrate degradation. In microglia, Ca^2+^ entry regulated podosome formation, migration and invasion. The podosomes contain Orai1, STIM1, and several Ca^2+^ responsive proteins: SK3, CaM and Iba1. In future, means of selectively targeting podosomes will be needed in order to determine if these structures are crucial for microglial migration and invasion through the brain.

## Abbreviations

2-APB: 2-aminoethyl diphenylborinate; Arp: Actin-related protein; BTP2: n-{4-[3,5-bis(trifluoromethyl)-1H-pyrazol-1-yl]phenyl}-4-methyl-1,2,3-thiadiazole-5-carboxamide; CaM: Calmodulin; CNS: Central nervous system; CRAC: Calcium release activated calcium; ECM: Extracellular matrix; EGTA: Ethyleneglycol-bis(β-aminoethyl)-N,N,N’,N’-tetraacetic acid; ER: Endoplasmic reticulum; F-actin: Filamentous actin; FBS: Fetal bovine serum; HEPES: 4-(2-hydroxyethyl)-1-piperazineethanesulfonic acid; Iba1: Ionized Ca^2+^ binding adaptor molecule 1; MEM: Minimal essential medium; Nox1: Nicotinamide adenine dinucleotide phosphate oxidase 1; PBS: Phosphate-buffered saline; SK: Small-conductance Ca^2+^-activated K^+^; SOCE: Store-operated Ca^2+^ entry; STIM1: Stromal interaction molecule 1; Tks5: Tyrosine kinase substrate with five Src homology 3 domains; TL: Tomato lectin; TRP: Transient receptor potential; WASp: Wiskott-Aldrich Syndrome protein.

## Competing interests

The authors declare that they have no competing interests.

## Authors’ contributions

TAS carried out the immunocytochemistry and analysis in Figures 2C, 2D, 3B, 3C, 4, 5, and 6. SL conducted and analyzed the migration, scratch-wound and invasion assays in Figure 7 and the immunocytochemistry in Figure 3A. CV did the staining in Figures 1, 2B, and 2E. LCS conceived and designed the project, obtained funding, supervised the work, and played a major role in interpreting results. LCS, TAS and SL prepared the manuscript. All authors read and approved the final manuscript.
